# Bis(ethyl­enediammonium) tetra­deca­borate

**DOI:** 10.1107/S1600536810008494

**Published:** 2010-03-13

**Authors:** Guo-Ming Wang, Pei Wang, Zeng-Xin Li, Hui Li, Hui-Luan Liu

**Affiliations:** a Department of Chemistry, Teachers College of Qingdao University, Qingdao, Shandong 266071, People’s Republic of China

## Abstract

The title compound, 2C_2_H_10_N_2_^2+^·B_14_O_20_(OH)_6_^4−^, consists of a centrosymmetric tetra­deca­borate anion and two ethyl­enediammonium cations. The anions are inter­connected through strong O—H⋯O hydrogen bonds into a three-dimensional supra­molecular network with channels along [100], [010], [001] and [111]. The diprotonated cations reside in the channels and inter­act with the inorganic framework by extensive N—H⋯O hydrogen bonds.

## Related literature

For general background to the structures and applications of inorganic borates, see: Burns *et al.* (1995 ▶); Chen *et al.* (1995 ▶); Grice *et al.* (1999 ▶); Touboul *et al.* (2003 ▶); Wang *et al.* (2007 ▶). For some typical examples of organically templated non-metal borates, see: Li *et al.* (2008 ▶); Liu *et al.* (2006 ▶); Pan *et al.* (2007 ▶); Wang *et al.* (2004 ▶). For two typical examples of crystalline aluminoborates, see: Wang *et al.* (2008*a* ▶,*b* ▶). 
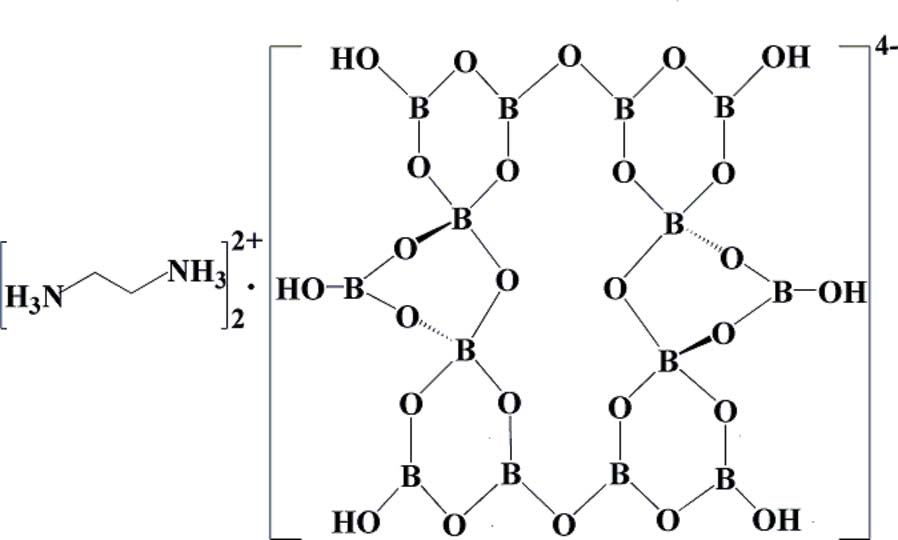


## Experimental

### 

#### Crystal data

2C_2_H_10_N_2_^2+^·B_14_H_6_O_26_^4−^
*M* *_r_* = 697.63
Triclinic, 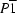

*a* = 8.4849 (3) Å
*b* = 8.8387 (3) Å
*c* = 10.0406 (2) Å
α = 95.085 (2)°
β = 96.942 (3)°
γ = 116.856 (4)°
*V* = 658.08 (3) Å^3^
*Z* = 1
Mo *K*α radiation
μ = 0.16 mm^−1^
*T* = 293 K
0.28 × 0.13 × 0.04 mm

#### Data collection

Bruker SMART APEX CCD diffractometer
Absorption correction: multi-scan (*SADABS*; Sheldrick, 1996 ▶) *T*_min_ = 0.956, *T*_max_ = 0.994
5101 measured reflections
2541 independent reflections
2033 reflections with *I* > 2σ(*I*)
*R*_int_ = 0.028

#### Refinement

*R*[*F* ^2^ > 2σ(*F* ^2^)] = 0.043
*wR*(*F* ^2^) = 0.107
*S* = 1.03
2541 reflections
217 parameters
H-atom parameters constrained
Δρ_max_ = 0.24 e Å^−3^
Δρ_min_ = −0.27 e Å^−3^

### 

Data collection: *SMART* (Bruker, 2007 ▶); cell refinement: *SAINT-Plus* (Bruker, 2007 ▶); data reduction: *SAINT-Plus*; program(s) used to solve structure: *SHELXS97* (Sheldrick, 2008 ▶); program(s) used to refine structure: *SHELXL97* (Sheldrick, 2008 ▶); molecular graphics: *SHELXTL* (Sheldrick, 2008 ▶) and *DIAMOND* (Brandenburg, 1999 ▶); software used to prepare material for publication: *SHELXTL*.

## Supplementary Material

Crystal structure: contains datablocks global, I. DOI: 10.1107/S1600536810008494/hy2288sup1.cif

Structure factors: contains datablocks I. DOI: 10.1107/S1600536810008494/hy2288Isup2.hkl

Additional supplementary materials:  crystallographic information; 3D view; checkCIF report

## Figures and Tables

**Table 1:** Hydrogen-bond geometry (Å, °)

*D*—H⋯*A*	*D*—H	H⋯*A*	*D*⋯*A*	*D*—H⋯*A*
O1—H1*F*⋯O8^i^	0.82	2.11	2.909 (2)	165
O8—H8*A*⋯O9^ii^	0.82	1.83	2.6433 (19)	176
O13—H13*A*⋯O7^iii^	0.82	1.79	2.6030 (18)	172
N1—H1*C*⋯O13^iv^	0.89	1.87	2.755 (2)	172
N1—H1*D*⋯O10^v^	0.89	2.04	2.919 (2)	168
N1—H1*E*⋯O2^v^	0.89	2.09	2.892 (2)	150
N2—H2*C*⋯O6^vi^	0.89	1.89	2.777 (2)	174
N2—H2*D*⋯O1^vii^	0.89	2.18	2.926 (2)	141
N2—H2*E*⋯O5^iii^	0.89	2.08	2.951 (2)	168

Symmetry codes: (i) 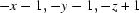; (ii) 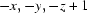; (iii) 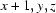; (iv) 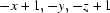; (v) 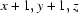; (vi) 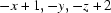; (vii) 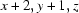.

**Fig. 1.**
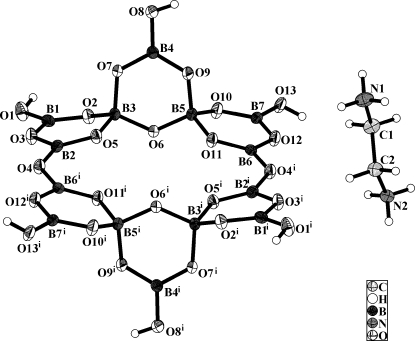
The molecular structure of the title compound. Displacement ellipsoids are drawn at the 50% probability level. [Symmetry code: (i) -x, -y, 2-z.]

**Fig. 2.**
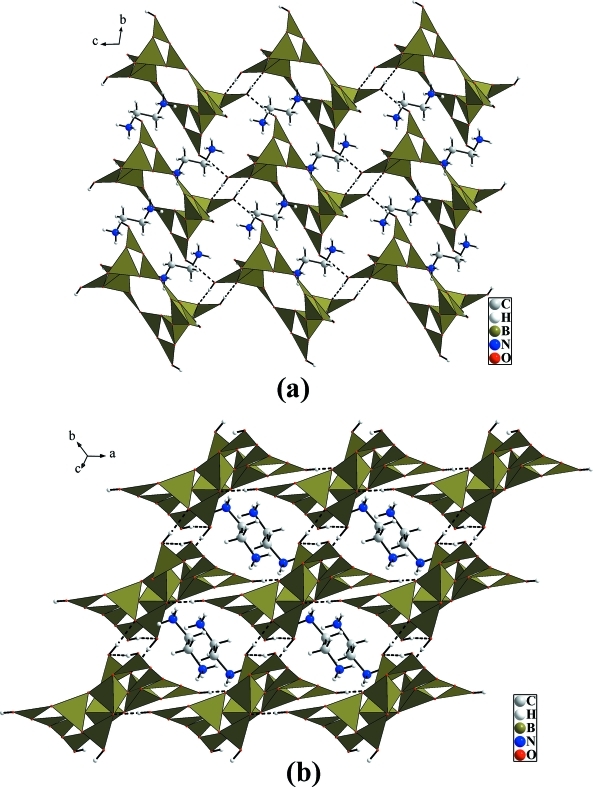
Different views (a) along [100] and (b) along [111] of the three-dimensional framework constructed from [B14O20(OH)6]4- anions, with [C2H10N2]2+ cations occupying channels. Hydrogen bonds are shown as dashed lines.

## supplementary crystallographic information

### Comment

Borate materials have attracted considerable attention in the past decades owing to their fascinating structural diversities and promising applications in mineralogy, luminescence and nonlinear optical properties (Burns *et al.*, 1995; Chen *et al.*, 1995; Grice *et al.*, 1999; Touboul *et al.*, 2003; Wang *et al.*, 2007). From a structural chemistry point of view, the ability of boron to adopt both BO_4_ and BO_3_ coordination modes, coupled with the tendency of such units to polymerize into a large range of polyanions, has made inorganic borates into a rapidly growing family. To date, borate materials with various alkali metal, alkaline earth metal, rare earth and transition metal, traditionally prepared under high temperature/pressure solid-state conditions, have been extensively studied. In contrast, the template synthesis of nonmetal borates is still a relatively undeveloped area. Recently, solvothermal method has been proved to be very effective in isolating such borates by employing various organic molecules as templates or structure-directing agents (Li *et al.*, 2008; Liu *et al.*, 2006; Pan *et al.*, 2007; Wang *et al.*, 2004). Our interest is to explore the introduction of aluminium into borate system, constructing novel microporous aluminoborate materials templated by organic agents with different shape and size (Wang *et al.*, 2008a, b). Interestingly, the title compound was obtained, which is a new organically templated nonmetal tetradecaborate.

As shown in Fig. 1, the asymmetric unit of the title compound consists of one [B_7_O_10_(OH)_3_]^2-^ anionic unit and one [C_2_H_10_N_2_]^2+^ cation. The anionic unit is composed of two BO_4_ tetrahedra [B3 and B5], two BO_3_ [B2 and B6] and three BO_2_(OH) [B1, B4 and B7] trigonal units, which forms three classic B_3_O_3_ cycles linked by two common BO_4_ tetrahedra. Two such [B_7_O_10_(OH)_3_]^2-^ units are further jointed together through the exocyclic O atoms [O4 and O4^i^, symmetry code: (i) -x, -y, 2-z], generating the FBBs (Fundamental Building Blocks), a large isolated [B_14_O_20_(OH)_6_]^4-^ polyanion. Thus, the borate FBBs, featuring one cyclic 8-membered ring (MR) and six 3-MRs, is made up of four BO_4_ and ten BO_3_ and BO_2_(OH) units. The B—O bond distances lie in the range 1.337 (3)–1.386 (3) Å for the BO_3_ triangles (av. 1.361 Å) and 1.430 (3)–1.489 (3) Å for the BO_4_ tetrahedra (av. 1.466 Å), in good agreement with those reported previously for other borate materials. The O—B—O bond angles of the BO_4_ tetrahedra lie in the range of 106.7 (2)–112.6 (2)° and those of the BO_3_ triangles span from 115.4 (2) to 123.4 (2)°; the averages for the corresponding angles are very close to 109.5 and 120°, respectively.

The FBBs, [B_14_O_20_(OH)_6_]^4-^, are connected with each other through strong intermolecular O—H···O hydrogen bonds (Table 1), forming a three-dimensional framework with channels along [100], [010], [001] and [111] directions. The diprotonated [C_2_H_10_N_2_]^2+^ cations reside in the channels, interacting with the framework through N—H···O hydrogen bonds (Fig. 2).

### Experimental

A mixture of H_3_BO_3_ (0.217 g), Al_2_O_3_ (0.104 g), ethylenediamine (0.42 ml), pyridine (5.0 ml) and H_2_O (0.90 ml) was sealed in a Teflon-lined steel autoclave, heated at 443 K for 10 d, and then cooled to room temperature. The colorless prism-shaped crystals were separated from the solution by filtration, washed with distilled water and dried in air.

### Refinement

All H atoms were positioned geometrically and treated as riding atoms, with O—H = 0.82, N—H = 0.89 and C—H = 0.97 Å and with *U*_iso_(H) = 1.2*U*_eq_(C,N) or 1.5*U*_eq_(O).

### Crystal data

**Table:**

2C_2_H_10_N_2_^2+^·B_14_H_6_O_26_^4^^−^	*Z* = 1
*M**_r_* = 697.63	*F*(000) = 356
Triclinic, *P*1	*D*_x_ = 1.760 Mg m^−^^3^
Hall symbol: -P 1	Mo *K*α radiation, λ = 0.71073 Å
*a* = 8.4849 (3) Å	Cell parameters from 5101 reflections
*b* = 8.8387 (3) Å	θ = 2.1–26.0°
*c* = 10.0406 (2) Å	µ = 0.16 mm^−^^1^
α = 95.085 (2)°	*T* = 293 K
β = 96.942 (3)°	Prism, colorless
γ = 116.856 (4)°	0.28 × 0.13 × 0.04 mm
*V* = 658.08 (3) Å^3^	

### Data collection

**Table:**

Bruker SMART APEX CCD diffractometer	2541 independent reflections
Radiation source: fine-focus sealed tube	2033 reflections with *I* > 2σ(*I*)
graphite	*R*_int_ = 0.028
φ and ω scans	θ_max_ = 26.0°, θ_min_ = 2.1°
Absorption correction: multi-scan (*SADABS*; Sheldrick, 1996)	*h* = −10→10
*T*_min_ = 0.956, *T*_max_ = 0.994	*k* = −10→10
5101 measured reflections	*l* = −12→12

### Refinement

**Table:**

Refinement on *F*^2^	Primary atom site location: structure-invariant direct methods
Least-squares matrix: full	Secondary atom site location: difference Fourier map
*R*[*F*^2^ > 2σ(*F*^2^)] = 0.043	Hydrogen site location: inferred from neighbouring sites
*wR*(*F*^2^) = 0.107	H-atom parameters constrained
*S* = 1.03	*w* = 1/[σ^2^(*F*_o_^2^) + (0.0518*P*)^2^ + 0.1264*P*] where *P* = (*F*_o_^2^ + 2*F*_c_^2^)/3
2541 reflections	(Δ/σ)_max_ < 0.001
217 parameters	Δρ_max_ = 0.24 e Å^−^^3^
0 restraints	Δρ_min_ = −0.27 e Å^−^^3^

### Fractional atomic coordinates and isotropic or equivalent isotropic displacement parameters (Å^2^)

**Table:**

	*x*	*y*	*z*	*U*_iso_*/*U*_eq_	
B1	−0.5849 (3)	−0.5170 (3)	0.8129 (2)	0.0190 (5)	
B2	−0.4605 (3)	−0.2456 (3)	0.9547 (2)	0.0180 (5)	
B3	−0.2914 (3)	−0.2948 (3)	0.7854 (2)	0.0166 (5)	
B4	−0.2007 (3)	−0.1473 (3)	0.5881 (2)	0.0166 (5)	
B5	0.0303 (3)	−0.1771 (3)	0.7443 (2)	0.0175 (5)	
B6	0.3485 (3)	0.0104 (3)	0.8520 (2)	0.0174 (5)	
B7	0.2775 (3)	−0.2278 (3)	0.6835 (2)	0.0189 (5)	
O1	−0.73216 (19)	−0.67207 (18)	0.76829 (15)	0.0286 (4)	
H1F	−0.7172	−0.7203	0.7011	0.043*	
O2	−0.42739 (18)	−0.47558 (17)	0.77218 (13)	0.0203 (3)	
O3	−0.60553 (17)	−0.40525 (18)	0.90605 (14)	0.0232 (3)	
O4	−0.48111 (18)	−0.15100 (18)	1.06075 (14)	0.0211 (3)	
O5	−0.31106 (17)	−0.18999 (17)	0.90041 (13)	0.0188 (3)	
O6	−0.11436 (17)	−0.27927 (17)	0.80925 (13)	0.0175 (3)	
O7	−0.32891 (17)	−0.23425 (18)	0.65880 (13)	0.0218 (3)	
O8	−0.25158 (18)	−0.10364 (18)	0.46895 (14)	0.0245 (4)	
H8A	−0.1620	−0.0404	0.4401	0.037*	
O9	−0.02594 (18)	−0.10073 (19)	0.63517 (14)	0.0246 (4)	
O10	0.10144 (17)	−0.28587 (18)	0.68168 (14)	0.0230 (3)	
O11	0.17413 (17)	−0.03462 (17)	0.84417 (13)	0.0196 (3)	
O12	0.40563 (17)	−0.08058 (18)	0.76988 (14)	0.0221 (3)	
O13	0.33259 (18)	−0.31501 (19)	0.59850 (14)	0.0248 (4)	
H13A	0.4399	−0.2832	0.6234	0.037*	
C1	0.8308 (3)	0.2448 (3)	0.7230 (2)	0.0251 (5)	
H1A	0.9116	0.2282	0.6699	0.030*	
H1B	0.7200	0.1376	0.7087	0.030*	
C2	0.9149 (3)	0.2927 (3)	0.8712 (2)	0.0248 (5)	
H2A	1.0184	0.4058	0.8868	0.030*	
H2B	0.8291	0.2977	0.9248	0.030*	
N1	0.7928 (3)	0.3808 (2)	0.67745 (18)	0.0284 (4)	
H1C	0.7433	0.3510	0.5897	0.043*	
H1D	0.8947	0.4789	0.6897	0.043*	
H1E	0.7173	0.3948	0.7254	0.043*	
N2	0.9717 (2)	0.1666 (2)	0.91544 (17)	0.0236 (4)	
H2C	1.0208	0.1977	1.0033	0.035*	
H2D	1.0518	0.1633	0.8673	0.035*	
H2E	0.8765	0.0631	0.9024	0.035*	

### Atomic displacement parameters (Å^2^)

**Table:**

	*U*^11^	*U*^22^	*U*^33^	*U*^12^	*U*^13^	*U*^23^
B1	0.0211 (12)	0.0190 (12)	0.0172 (12)	0.0090 (10)	0.0043 (10)	0.0053 (10)
B2	0.0159 (11)	0.0207 (12)	0.0179 (11)	0.0094 (9)	0.0017 (9)	0.0020 (9)
B3	0.0139 (11)	0.0185 (11)	0.0171 (11)	0.0069 (9)	0.0044 (9)	0.0032 (9)
B4	0.0171 (11)	0.0167 (11)	0.0167 (11)	0.0083 (9)	0.0042 (9)	0.0019 (9)
B5	0.0131 (11)	0.0209 (12)	0.0183 (12)	0.0076 (9)	0.0030 (9)	0.0035 (9)
B6	0.0175 (11)	0.0228 (12)	0.0138 (11)	0.0103 (10)	0.0045 (9)	0.0054 (9)
B7	0.0158 (11)	0.0266 (12)	0.0158 (11)	0.0112 (10)	0.0024 (9)	0.0036 (10)
O1	0.0228 (8)	0.0213 (8)	0.0322 (9)	0.0024 (6)	0.0106 (7)	−0.0046 (7)
O2	0.0184 (7)	0.0178 (7)	0.0222 (8)	0.0061 (6)	0.0065 (6)	0.0002 (6)
O3	0.0155 (7)	0.0220 (8)	0.0262 (8)	0.0042 (6)	0.0069 (6)	−0.0024 (6)
O4	0.0150 (7)	0.0232 (8)	0.0213 (8)	0.0072 (6)	0.0033 (6)	−0.0041 (6)
O5	0.0158 (7)	0.0186 (7)	0.0197 (7)	0.0063 (6)	0.0051 (6)	−0.0010 (6)
O6	0.0141 (7)	0.0220 (7)	0.0173 (7)	0.0085 (6)	0.0040 (6)	0.0051 (6)
O7	0.0133 (7)	0.0334 (8)	0.0201 (7)	0.0107 (6)	0.0048 (6)	0.0096 (6)
O8	0.0175 (7)	0.0308 (8)	0.0231 (8)	0.0081 (6)	0.0045 (6)	0.0115 (6)
O9	0.0146 (7)	0.0334 (8)	0.0254 (8)	0.0085 (6)	0.0063 (6)	0.0147 (7)
O10	0.0150 (7)	0.0255 (8)	0.0251 (8)	0.0086 (6)	0.0020 (6)	−0.0056 (6)
O11	0.0141 (7)	0.0216 (7)	0.0221 (8)	0.0085 (6)	0.0036 (6)	−0.0025 (6)
O12	0.0140 (7)	0.0267 (8)	0.0233 (8)	0.0091 (6)	0.0027 (6)	−0.0045 (6)
O13	0.0149 (7)	0.0349 (9)	0.0233 (8)	0.0133 (7)	−0.0001 (6)	−0.0064 (7)
C1	0.0281 (12)	0.0204 (11)	0.0263 (12)	0.0123 (9)	0.0008 (9)	0.0005 (9)
C2	0.0277 (12)	0.0251 (12)	0.0238 (12)	0.0141 (10)	0.0047 (9)	0.0039 (9)
N1	0.0355 (11)	0.0266 (10)	0.0234 (10)	0.0175 (9)	−0.0022 (8)	−0.0021 (8)
N2	0.0239 (10)	0.0296 (10)	0.0188 (9)	0.0133 (8)	0.0046 (7)	0.0054 (8)

### Geometric parameters (Å, °)

**Table:**

B1—O2	1.343 (3)	B7—O10	1.340 (3)
B1—O1	1.360 (3)	B7—O13	1.359 (3)
B1—O3	1.383 (3)	B7—O12	1.386 (3)
B2—O5	1.341 (3)	O1—H1F	0.8200
B2—O4	1.372 (3)	O4—B6^i^	1.372 (3)
B2—O3	1.381 (3)	O8—H8A	0.8200
B3—O6	1.433 (3)	O13—H13A	0.8200
B3—O2	1.471 (3)	C1—N1	1.474 (3)
B3—O7	1.476 (3)	C1—C2	1.503 (3)
B3—O5	1.489 (3)	C1—H1A	0.9700
B4—O7	1.344 (3)	C1—H1B	0.9700
B4—O9	1.355 (3)	C2—N2	1.481 (3)
B4—O8	1.369 (3)	C2—H2A	0.9700
B5—O6	1.430 (3)	C2—H2B	0.9700
B5—O11	1.474 (3)	N1—H1C	0.8900
B5—O9	1.477 (3)	N1—H1D	0.8900
B5—O10	1.480 (3)	N1—H1E	0.8900
B6—O11	1.337 (3)	N2—H2C	0.8900
B6—O4^i^	1.372 (3)	N2—H2D	0.8900
B6—O12	1.376 (3)	N2—H2E	0.8900
			
O2—B1—O1	122.44 (19)	B5—O6—B3	125.67 (16)
O2—B1—O3	121.71 (19)	B4—O7—B3	122.80 (16)
O1—B1—O3	115.84 (18)	B4—O8—H8A	109.5
O5—B2—O4	123.39 (19)	B4—O9—B5	122.54 (16)
O5—B2—O3	121.17 (18)	B7—O10—B5	121.89 (17)
O4—B2—O3	115.44 (18)	B6—O11—B5	123.54 (16)
O6—B3—O2	110.41 (17)	B6—O12—B7	118.51 (17)
O6—B3—O7	112.48 (16)	B7—O13—H13A	109.5
O2—B3—O7	106.77 (16)	N1—C1—C2	110.38 (17)
O6—B3—O5	109.26 (16)	N1—C1—H1A	109.6
O2—B3—O5	109.91 (16)	C2—C1—H1A	109.6
O7—B3—O5	107.96 (16)	N1—C1—H1B	109.6
O7—B4—O9	120.90 (18)	C2—C1—H1B	109.6
O7—B4—O8	117.95 (18)	H1A—C1—H1B	108.1
O9—B4—O8	121.13 (18)	N2—C2—C1	111.20 (17)
O6—B5—O11	110.14 (16)	N2—C2—H2A	109.4
O6—B5—O9	112.55 (16)	C1—C2—H2A	109.4
O11—B5—O9	107.35 (16)	N2—C2—H2B	109.4
O6—B5—O10	109.49 (17)	C1—C2—H2B	109.4
O11—B5—O10	109.92 (16)	H2A—C2—H2B	108.0
O9—B5—O10	107.33 (16)	C1—N1—H1C	109.5
O11—B6—O4^i^	122.59 (19)	C1—N1—H1D	109.5
O11—B6—O12	121.46 (19)	H1C—N1—H1D	109.5
O4^i^—B6—O12	115.93 (18)	C1—N1—H1E	109.5
O10—B7—O13	119.34 (19)	H1C—N1—H1E	109.5
O10—B7—O12	121.74 (19)	H1D—N1—H1E	109.5
O13—B7—O12	118.91 (18)	C2—N2—H2C	109.5
B1—O1—H1F	109.5	C2—N2—H2D	109.5
B1—O2—B3	120.65 (17)	H2C—N2—H2D	109.5
B2—O3—B1	118.42 (16)	C2—N2—H2E	109.5
B2—O4—B6^i^	127.25 (17)	H2C—N2—H2E	109.5
B2—O5—B3	122.53 (16)	H2D—N2—H2E	109.5

Symmetry codes: (i) −*x*, −*y*, −*z*+2.

### Hydrogen-bond geometry (Å, °)

**Table:**

*D*—H···*A*	*D*—H	H···*A*	*D*···*A*	*D*—H···*A*
O1—H1F···O8^ii^	0.82	2.11	2.909 (2)	165
O8—H8A···O9^iii^	0.82	1.83	2.6433 (19)	176
O13—H13A···O7^iv^	0.82	1.79	2.6030 (18)	172
N1—H1C···O13^v^	0.89	1.87	2.755 (2)	172
N1—H1D···O10^vi^	0.89	2.04	2.919 (2)	168
N1—H1E···O2^vi^	0.89	2.09	2.892 (2)	150
N2—H2C···O6^vii^	0.89	1.89	2.777 (2)	174
N2—H2D···O1^viii^	0.89	2.18	2.926 (2)	141
N2—H2E···O5^iv^	0.89	2.08	2.951 (2)	168

Symmetry codes: (ii) −*x*−1, −*y*−1, −*z*+1; (iii) −*x*, −*y*, −*z*+1; (iv) *x*+1, *y*, *z*; (v) −*x*+1, −*y*, −*z*+1; (vi) *x*+1, *y*+1, *z*; (vii) −*x*+1, −*y*, −*z*+2; (viii) *x*+2, *y*+1, *z*.
